# Single-cell analysis of cell viability after a biocide treatment unveils an absence of positive correlation between two commonly used viability markers

**DOI:** 10.1002/mbo3.62

**Published:** 2012-12-26

**Authors:** Adrien Ducret, Sam Dukan

**Affiliations:** Laboratoire de Chimie Bactérienne, Institut de Microbiologie de la Méditerranée – Université Aix-Marseille, CNRS UMR728331 Chemin Joseph Aiguier, Marseille, 13009, France

**Keywords:** CV6, DVC, *E. coli*, hypochlorous acid, ibidi chamber

## Abstract

Discrimination among viable/active or dead/inactive cells in a microbial community is a vital question to address issues on ecological microbiology or microbiological quality control. It is commonly assumed that metabolically active cells (ChemchromeV6 [CV6] procedure) correspond to viable cells (direct viable count procedure [DVC]), although this assumption has never been demonstrated and is therefore a matter of debate. Indeed, simultaneous determination of cell viability and metabolic activity has never been performed on the same cells. Here, we developed a microfluidic device to investigate the viability and the metabolic activity of *Escherichia coli* cells at single-cell level. Cells were immobilized in a flow chamber in which different solutions were sequentially injected according to different scenarios. By using time-lapse microscopy combined with automated tracking procedures, we first successfully assessed the ability of cells to divide and their metabolic activity at single-cell level. Applying these two procedures on the same cells after a hypochlorous acid (HOCl) treatment, we showed that the ability of cells to divide and their metabolic activity were anticorrelated. These results indicate that the relation between CV6 uptake and cell viability may be partially incorrect. Care must be taken in using the terms “CV6-positive” and “viable” synonymously.

## Introduction

In environmental microbiology, discrimination among viable or dead and active or inactive cells in a microbial community is a vital question to address issues on ecological microbiology, food safety, drinking water quality, infections of pathogens, or efficacy of disinfectants and antibiotics. Since the end of the 19th century, detection of viable bacteria has been carried out by cultivation and enumeration of colony forming units (CFU). Ability of cells to grow in selective media and form colonies on nutrient agar plates is routinely used as a retrospective criterion for the detection of viable bacteria. Nonetheless, the reliability of culture methods often depends on bacterial species and media composition, leading to an underestimation of viable cell counts (Dukan et al. 1999; Gogniat et al. 2006; Cuny et al. 2007; Oliver 2010). Hence, the latest characterization of the “viable but non culturable” state definitely renders the ability to grow on agar plates inappropriate to assess the cell viability (Oliver 2010).

New methods have been developed over the last decades to address the viability of cells. The most commonly employed methods include single-cell-based assays targeting reliable metabolic activities (Kogure et al. 1979; Joux and Lebaron 1997; Parthuisot et al. 2000; Delgado-Viscogliosi et al. 2005) (e.g., esterase activity, membrane potential, DNA synthesis, respiration…), membrane integrity (Berney et al. 2007), or the ability to divide (Joux and Lebaron 1997), by combining fluorescent reporters and fluorescence microscopy or flow cytometry.

Among these methods, the procedure based on the CV6 assay is commonly performed for the detection of metabolically active cells in a large range of conditions, like for water quality assessment (Parthuisot et al. 2000; Johnson et al. 2006; Alleron et al. 2008). CV6 are fluorogenic esters, which are converted to free fluorescein by esterase activity of viable cells. The concentration of fluorescein trapped in metabolically active cells increases over time as a function of esterase activity. In contrast to inactive cells, which did not show any green fluorescence (CV6-negative), cells that are brightly stained with CV6 can be characterized as viable (CV6-positive).

By comparison, instead of observing only a specific metabolic activity of individual cells (Kogure et al. 1979; Joux and Lebaron 1997), methods such as the direct viable count (DVC) procedure have been developed to assess the ability of cells to divide. Using the DVC procedure, cells are incubated with nutrients in the presence of an antibiotic, which prevents cell division (Kogure et al. 1979; Joux and Lebaron 1997). Only cells which are able to divide will continue to metabolize nutrients and become elongated after incubation. As cell elongation implies that the cell (1) has an intact membrane, (2) is metabolically active, and (3) is able to grow in the presence of nutrients, it is commonly assumed that the DVC procedure is one of the most integrative procedures to estimate the cell viability at single-cell level, up to date.

Due to easiest implementation, viability tests targeting only a specific metabolic activity are more routinely used in microbial ecology than more integrative tests like the DVC procedure. However, the assumption that metabolically active cells (CV6-positive) correspond to viable cells has never been demonstrated and is still a matter of debate. For this reason and for the rest of this manuscript, we will use “metabolically active cells” and not “viable” cells for CV6-positive cells.

Here, using a microfluidic device, we set up a system to determine the metabolic activity (CV6) and the ability to divide (DVC) on the same cells. *Escherichia coli* cells were immobilized in a flow chamber in which different solutions including media, fluorescent dyes, and biocide were sequentially injected according to different scenarios. Using time-lapse microscopy combined with automated tracking procedures, we first successfully assessed the ability of cells to divide (DVC) and their metabolic activity (CV6) at single-cell level at an unprecedented degree of characterization. The lag and apparent doubling times were estimated individually for each cell using DVC procedure as well as the accumulation profile of fluorescent dye using the CV6 procedure. Applying these two procedures on the same cells after a biocide treatment (hypochlorous acid [HOCl]), we found that the ability of cells to divide and the metabolic activity were differentially affected by a HOCl treatment and consequently that the cell viability estimated using DVC and CV6 were unexpectedly anticorrelated.

## Experimental Procedures

### Bacterial strains and growth

*Escherichia coli* K-12 (MG1655) strain was grown aerobically in liquid Luria–Bertani (LB) medium in a rotary shaker at 37°C and 160 rpm.

### Microfluidic system and microscopy

To immobilize the cells, the μ-Slide chambers were functionalized using poly-l-lysine as previously described (Dhouib et al. 2011). Cells were harvested during the exponential growth phase (OD_600_ = 0.5), washed twice by centrifugation in Phosphate-Buffered Saline (PBS, pH 7.4, 10 mmol/L), and then immobilized in the functionalized μ-Slide chamber (Ibidi©, Martinsried, Germany). The μ-Slide chamber was centrifuged (2 min at 1500 g) to enhance cell adhesion and each flow channel was then directly connected to injectors.

### Data acquisition and analysis

Microscopic analysis was performed using an automated and inverted epifluorescence microscope TE2000-E-PFS (Nikon, Champigny Sur Marne, France). The microscope is equipped with the “Perfect Focus System” (PFS) that automatically maintains focus so that the point of interest within a specimen is always kept in sharp focus at all times, in spite of any mechanical or thermal perturbations. For each experiment, 10 separate fields containing at least 20 cells each, were manually or automatically defined with a motorized stage (Prior Scientific, Cambridge, U.K.) and stored (X, Y, Z, PFS-offset) in our custom automation system designed for time-lapse experiments. The microscope is placed within an incubator that allows precise regulation of the external temperature (37°C). Images were recorded using a CoolSNAP HQ 2 (Roper Scientific SARL, Evry, France) and a 40×/0.75 DLL “Plan-Apochromat” or a 100×/1.4 DLL objective. Resulting images have spatial dimensions of 0.16 μm/pixel and 0.064 μm/pixel respectively. Excitation light was emitted by a 120-W metal halide light and signals were monitored using appropriate filters. All fluorescence images were acquired with a minimal exposure time to minimize bleaching and phototoxicity effects. As a control, a field that was not exposed to fluorescent illumination was monitored in similar conditions. Time-lapse experiments, digital analysis, and image processing were conducted by a custom automation script (Visual Basic) under Metamorph 7.5 (Molecular Devices, St. Grégoire, France). Images were processed and analyzed as previously described (Ducret et al. 2009). Elongation ratio was calculated for each cell as follows: elongation ratio = (maximal cell length − initial cell length)/initial cell length. Accumulation ratio was calculated for each cell as follows: accumulation ratio = (maximal cell fluorescence intensity − initial cell fluorescence intensity)/initial cell fluorescence intensity.

### Cell viability and activity assessments

Assessment of cell viability of *E. coli* was followed using CV6 procedure (ChemChrome V6; CV6 – AES-Chemunex, Ivry-Sur-Seine, France) and/or DVC procedure (Kogure et al. 1979; Joux and Lebaron 1997). CV6 labeling solution was prepared according to the manufacturer's instructions. Briefly, 10 μL of CV6 (#200-R1007-03) were diluted in 1 mL of PBS pH 7.4. The CV6 solution was added at 1 μL/sec in the flow chamber for 10 min in the dark at 37°C. Cells were rinsed by injection of PBS pH 7.4 (1 μL/sec). DVC procedure was performed by injecting 10 mL of LB supplemented with ciprofloxacin (4 mg/L) at 1 μL/sec in the flow chamber. Cells were incubated for 3 h at 37°C.

### HOCl treatment

Fresh solution of sodium hypochlorite was prepared daily in distilled water and used immediately. NaClO solution in distilled water was stable for several hours. We use the term HOCl throughout the article to include both undissociated acid and hypochlorite ion. For HOCl treatment, HOCl was added at 0.05 mg/L in PBS pH 7.4 and then injected at 1 μL/sec in the flow chamber for 10 min in the dark at 37°C. Cells were rinsed by injection of PBS pH 7.4 (1 μL/sec).

## Results and Discussion

### The DVC procedure can be used at the single-cell level in a flow chamber

**Figure 1:**
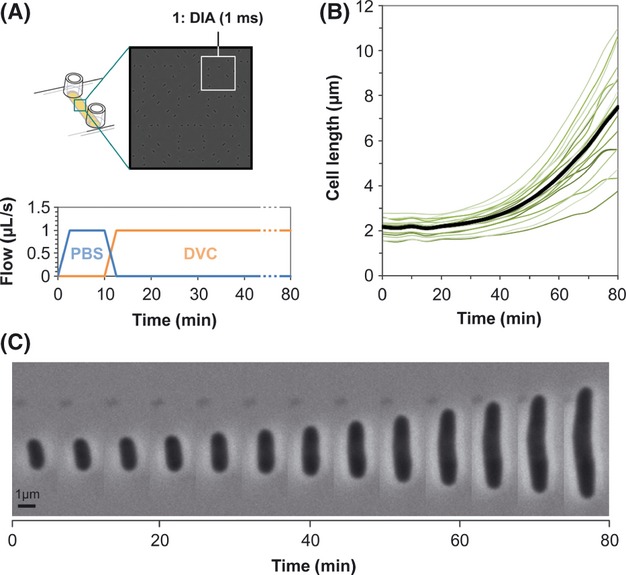
Single-cell analysis of cell viability using direct viable count (DVC) procedure. (A) Layout of the system and injection schedule used for DVC experiments. (B) Quantification of the cell length (*n* = 30) as a function of time (min). The dark line gives the average value of the population observed. (C) Sequence of images showing several rounds of cell elongation as a function of time (min). Scale bar = 1 μm.

To assess the viability of immobilized cells, we first tried to allow cells, harvested from a liquid exponential phase growth culture, to develop under a constant stream of LB. Unfortunately, after cell division, daughter cells were detached from the substrate and were lost in the flow, thus precluding any quantitative analysis of cell viability as a measure of growth and the formation of a microcolony (data not shown). To overcome this limitation, we tested whether the DVC procedure could be performed in this flow chamber. As previously described, the DVC procedure is based on the incubation of cells with nutrients in the presence of an antibiotic that prevents cell division (Kogure et al. 1979; Joux and Lebaron 1997). After incubation, viable cells become elongated. Immobilized cells were first incubated in PBS for 10 min and then observed under a constant stream of LB supplemented with ciprofloxacin for 70 min at 37°C and their length was measured over the time (Fig. 1A). After Incubation, 98% of the cells were elongated and the average cell length increased from 2.18 ± 0.33 to 7.49 ± 2.15 μm (Fig. 1B and C; Movie S1). As observed in Figure S1, the elongation rate of DVC-treated cells and nontreated cells under an agar pad are similar even after a cell division event. In these conditions, the apparent doubling times can be estimated individually for each DVC-treated cell as the time required to observe a twofold increase of the cell length. In the absence of exogenous stress, the average lag time was 19.2 ± 4.8 min and the apparent doubling time was 24.7 ± 2.7 min, which are fairly similar to the lag and doubling times of dividing cells observed in LB liquid culture or under an agar pad (Ducret et al. 2009) (Figure S1). As a result, the DVC procedure can be used to estimate the viability of individual cells in the microfluidic chamber.

### Metabolic activity can be estimated at the single-cell level in a flow chamber using the CV6 procedure

**Figure 2:**
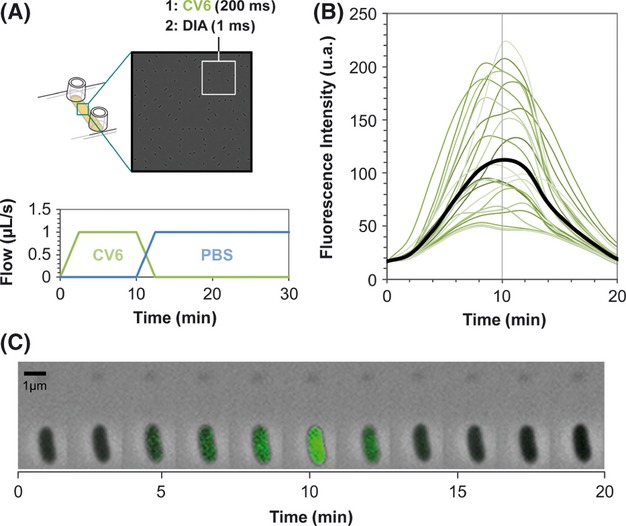
Single-cell analysis of cell activity using ChemchromeV6 (CV6) procedure. (A) Layout of the system and injection schedule used for CV6 experiments. (B) Quantification of the cell fluorescence intensity (*n* = 30) as a function of time (min). The dark line gives the average value of the population observed. (C) Sequence of images showing several rounds of cell stained by CV6 as a function of time (min). Phase images and fluorescent signal are overlaid. Scale bar = 1 μm.

We next tested whether metabolic activity could be estimated at the single-cell level in a microfluidic device with the CV6 procedure (Parthuisot et al. 2000; Delgado-Viscogliosi et al. 2005). A CV6 solution was injected for 10 min and then rinsed by a continuous flow of PBS (Fig. 2A). During the time course of the experiment, cells were illuminated 200 msec every 2 min using the appropriate filters. As expected, the average fluorescence intensity of stained cells increased from 16.6 ± 0.9 arbitrary units (a.u.) to 223.7 ± 51.3 a.u. when the CV6 solution was injected (Fig. 2B and C; Movie S2). Computed accumulation rates ranged between 9.1 and 23.5 a.u./min, suggesting that a subset of cells were more metabolically active than the rest of the population. Strikingly, the fluorescence emitted by cells decreased after cells were rinsed with PBS. Due to their negative charge at neutral pH, fluorescent molecules should be maintained inside the cell by an intact membrane potential. But, active dye extrusion by efflux pump, which had previously been observed (Breeuwer et al. 1994), suggests that the concentration of fluorescent molecules inside the cell is the result of an equilibrium between dye cleavage and dye extrusion.

Moreover, frequent and/or strong illuminations can generate a stress (phototoxicity) that may affect the viability of stained cells for the rest of the experiment. To test the effect of illumination on the viability of stained cells, we decided to conduct two consecutive sets of CV6 and PBS injections on the same immobilized cells (Fig. S2A). However, for the time course of the experiment, at least 100 cells were illuminated 200 msec (high illumination level) when 100 other cells were illuminated only 20 msec (low illumination level) every 2 min using the appropriate filters. In both cases, the average fluorescence intensity of stained cells first increased and then decreased when the CV6 solution was, respectively, injected and rinsed (Fig. S2C and D). However, whereas the accumulation rates of cells exposed at low illumination level (20 msec) were similar and linearly correlated to the accumulation rates observed during the first injection of CV6, these rates were clearly affected when cells were exposed at high illumination levels (200 msec) (Fig. S2B, C, and D). Altogether, these results suggest that metabolic activity can be estimated at the single-cell level using CV6 procedure in the microfluidic chamber. However, excessive illumination affects cell viability and may thus interfere with the fate of immobilized cells. Hence, to avoid viability assessment bias, cells have to be gently illuminated when stained with a fluorescent dye solution. Low illumination level (20 msec) was thus selected for further experiments. Finally, careful analyses of fluorescence profiles address two aspects of cell metabolism: the esterase activity and the activity of the efflux pump.

### The ability of cells to divide and metabolic activity were not affected at the same level by a moderate HOCl treatment

Next, we tested how the ability of cells to divide and the metabolic activity were affected by a moderate oxidative stress. For this purpose, and as indicated in the Experimental Procedures section, we used both procedures (CV6 and DVC) to assess the cell viability after a moderate HOCl treatment (0.05 mg/L). As an internal control, metabolic activity of each cell was also assessed before HOCl treatment.

**Figure 3:**
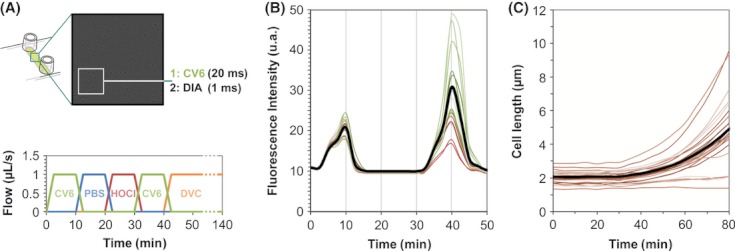
Single-cell analysis of cell viability and cell metabolic activity using ChemchromeV6 (CV6) and direct viable count (DVC) procedures before and/or after a moderate hypochlorous acid (HOCl) treatment (0.05 mg/L). (A) Layout of the system and injection schedule used for CV6/DVC/HOCl experiments. (B) Quantification of the cell fluorescence intensity (*n* = 30) as a function of time (min) before (t_0–20 min_) and after (t_30–50 min_) HOCl treatment. The cells showing a maximal accumulation rate higher after HOCl treatment than before were represented with green lines. In contrast, the cells showing a maximal accumulation rate after treatment that was similar to or lower than before are represented with red lines. The dark line gives the average value. (C) Quantification of the cell length (*n* = 30) as a function of time (min) after HOCl treatment.

As observed above, fluorescence intensity increased when the CV6 solution was injected and decreased when CV6 solution was rinsed (Fig. 3B). Strikingly, accumulation rates were not reduced by exposure to HOCl as expected, but were significantly higher than those observed before treatment. Most cells (85%) harbored a maximal value of fluorescence intensity 1.2- to 2.8-fold higher after HOCl exposure (Fig. 3B, green lines). As proteins are likely to be major targets for reaction with HOCl within a cell (Hawkins et al. 2003), we expected esterase activity to decrease after HOCl treatment. However, as shown above, the dye accumulation rate results not only from dye cleavage but also from passive dye uptake and active dye extrusion. Thus, the accumulation of fluorescence observed after HOCl treatment could result from a faster dye uptake or a slower dye extrusion. The cell membrane could become more permeable after a HOCl treatment leading to a faster uptake of uncleaved dye. However, the uncleaved form of CV6 is a carboxyfluorescein diacetate that diffuses freely through the cell membrane. Until its conversion to free fluorescein by cytoplasmic esterase, the uncleaved form passively diffuses both into and out of the cell. Thus, when CV6 solution is injected into the flow chamber, the concentration of uncleaved dye inside stressed or unstressed cells is never limiting. Interestingly, fluorescence decays observed when the CV6 solution was rinsed were significantly reduced after HOCl treatment (Fig. S3). Profiles of extrusion after the HOCl exposure were characterized by a half-life twofold higher than before (t_1/2_ from 0.89 ± 0.13 to 1.64 ± 0.4 min) or by a lag time that appeared before the exponential decay (Fig. S3), suggesting that active dye extrusion activity is reduced. Slowing down of active dye extrusion, instead of faster uptake, likely explains why accumulation rates increased after the HOCl exposure. Interestingly, it had previously been described that HOCl alters bacterial ATP production (Barrette et al. 1989). Activity of inner membrane-bound systems responsible of bacterial ATP production by both oxidative and fermentative pathways declined drastically after an HOCl treatment (Barrette et al. 1989). As a consequence, our results suggest that HOCl directly or indirectly affects the ATP-dependent activity of efflux pumps responsible for dye extrusion.

A minority of cells (15%) showed therefore similar accumulation rates (<10% of variation) before and after the HOCl treatment (Fig. 3B, red lines), suggesting that among the population observed, a subpopulation of cells was not or weakly affected by the HOCl treatment in terms of dye extrusion.

As observed in Figure 3C, the ability of immobilized cells to divide was also affected by the HOCl treatment. In contrast to nontreated cells, the average lag time and average doubling time increased from 19.2 ± 4.8 to 44.4 ± 20 min and from 24.7 ± 2.7 to 38.6 ± 6.9 min, respectively. Interestingly, the range between the minimal and the maximal cell length is higher after HOCl treatment (from 1.9 to 9.6 μm) than observed in nontreated cells (from 3.7 to 11.0 μm). Altogether, these results support the idea that the cell metabolic activity and the ability of cells to divide are not both negatively impacted by HOCl treatment as initially expected.

### Cell viability estimated using DVC and CV6 are anticorrelated after a moderate HOCl treatment

**Figure 4:**
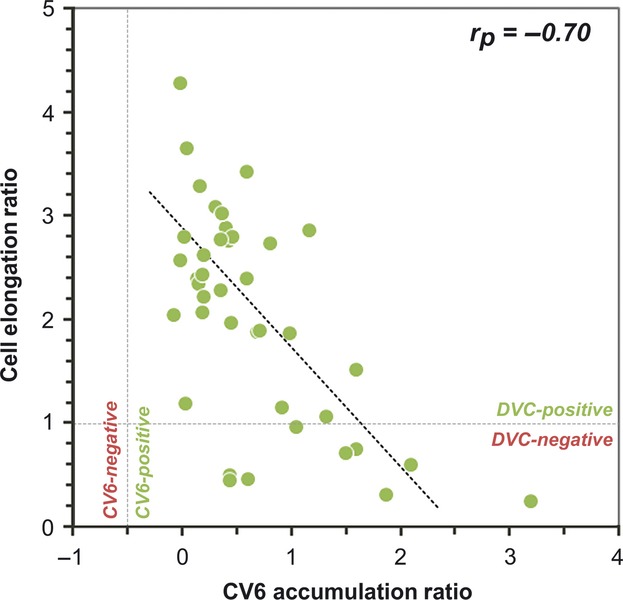
Cell viability estimated using direct viable count and ChemchromeV6 were not correlated. The relationship between the accumulation ratio and the elongation ratio observed after hypochlorous acid (HOCl) treatment. Vertical and horizontal light gray dashed lines represent the threshold usually used to discriminate CV6-positive/negative cells (vertical) or DVC-positive/negative cells (dashed line).

It is commonly accepted that brightly stained cells with CV6 are considerate as viable (CV6-positive). By assumption, the level of cell viability estimated using the CV6 procedure through the fluorescence accumulation ratio (see Experimental Procedures) should be positively correlated with the level of cell viability estimated using the DVC procedure. We observed therefore that the accumulation of CV6 fluorescence after a moderate HOCl treatment results primarily from a depletion of efflux pumps activity responsible for dye extrusion and consequently may indicate an alteration of cell viability. To definitively test the relationship between these two traits of cell viability, we compare the CV6-fluorescence accumulation ratio and the cell length elongation ratio for each cell after the moderate HOCl treatment. Surprisingly, and as depicted in Figure 4C, these two variables were anticorrelated (Pearson Coefficient: −0.70). Cells appearing as brightly stained with CV6 after an HOCl treatment (assumed to be CV6-positive) appear to be the most affected in terms of cell elongation (assumed to be DVC-negative). These results suggest that the CV6 procedure can lead to a misinterpretation of cell viability under the conditions used here.

The Chemchrome V6 procedure is a well-established viability procedure that has been used in a large range of conditions (Parthuisot et al. 2000; Johnson et al. 2006; Alleron et al. 2008). Basically, the efficiency of CV6 dyes to stain viable bacterial cells in water is based on the differential uptake of dye between “live” and artificially killed cells. Killed cells are usually obtained for this purpose by heat treatment (Delgado-Viscogliosi et al. 2005), ethanol treatment (Davey et al. 2004), or strong chlorine treatment (Delgado-Viscogliosi et al. 2005). However, such treatments that are designed to artificially reduce the viability of a control population are unlikely to be representative of the damage caused by less severe environmental conditions. We show here that under a moderate HOCl treatment, cells harboring high values of fluorescence, conventionally interpreted as metabolically active (Parthuisot et al. 2000, 2011), appear to be the most affected in terms of cell elongation by a moderate biocide treatment. In other words, the assumption that CV6 uptake indicates that cell is viable may be partially incorrect. Interestingly, similar misinterpretations are described when propidium iodide (PI) has been used to discriminate yeast cells with an intact or a damaged membrane (Davey and Hexley 2011). It is commonly assumed that the loss of membrane integrity represents irreparable damage and thus cell death. However, for moderate stress levels, a subpopulation of cells can take up PI immediately following exposure to stress, but retains the ability to repair the membrane damage such that subsequent exposure to PI does not result in staining (Davey and Hexley 2011). Altogether, these findings indicate that care must be taken regarding the terms used to describe CV6-positive, PI-positive, and viable cells.

Finally, we have demonstrated that single-cell analysis using microfluidics, in contrast to population-based experiments, is a powerful tool that yields insight into the characterization of viability at the cellular level. By using various injection scenarios and several strains expressing fluorescent fusion proteins, it is now possible to study the effect of many different stresses on the fate of every single cell in a population, in terms of viability or physiological traits (e.g., variations in gene expression).

## Supporting Information

**Figure S1.** Cell elongation of DVC treated and non-treated cells. (A) Sequence of images showing several rounds of cell elongation as a function of time (min) in presence or in absence of ciprofloxacin (4 mg/L). Scale bar = 1 μm. (B) Quantification of the cell length as a function of time (min). The green line gives the cell length of the DVC treated cells. The red line gives the cumulated cell length of the non-treated cells. The dashed red lines give the cell length of the two daughter cells.

**Figure S2.** Effect of illumination on cell viability using two consecutive sets of CV6 and PBS injections on the same immobilized cells. (A) Layout of the system and injection schedule used for two consecutive sets of CV6 injection. (B) The relationship between the accumulation ratio observed during the first injection of CV6 (T_10 min_) and the second injection of CV6 (T_30 min_), when cells were exposed 200 msec (left panel) or 20 msec (right panel). (C, D) Quantification of the cell fluorescence intensity (*n* = 30) as a function of time (min) when cells were exposed 200 msec (C) or 20 msec (D). The dark line gives the average value of the population observed.

**Figure S3.** CV6 extrusion profiles were significantly affected by HOCl. (A, B) Quantification of the cell fluorescence intensity (*n* = 30) as a function of time (min) before HOCl treatment (A) and after HOCl treatment (B). The dark line gives the average value of the population observed.

**Movie S1.** Cell elongation of DVC treated cells. Sequence of images showing several rounds of cell elongation as a function of time in presence of ciprofloxacin (4 mg/L). Images were taken every 2 min.

**Movie S2.** Single-cell analysis of cell activity using ChemchromeV6 (CV6) procedure. Sequence of images showing several rounds of cells stained by CV6 as a function of time. Phase images are on the left panel and fluorescent signal are on the right panel. Images were taken every 2 min.
