# Supplementary figures and images for: Single-cell analysis of cell viability after a biocide treatment unveils an absence of positive correlation between two commonly used viability markers
Source: Microbiologyopen. 2012 Dec 26;2(1):123–9. doi: 10.1002/mbo3.62 (PMC3584218; doi:10.1002/mbo3.62)

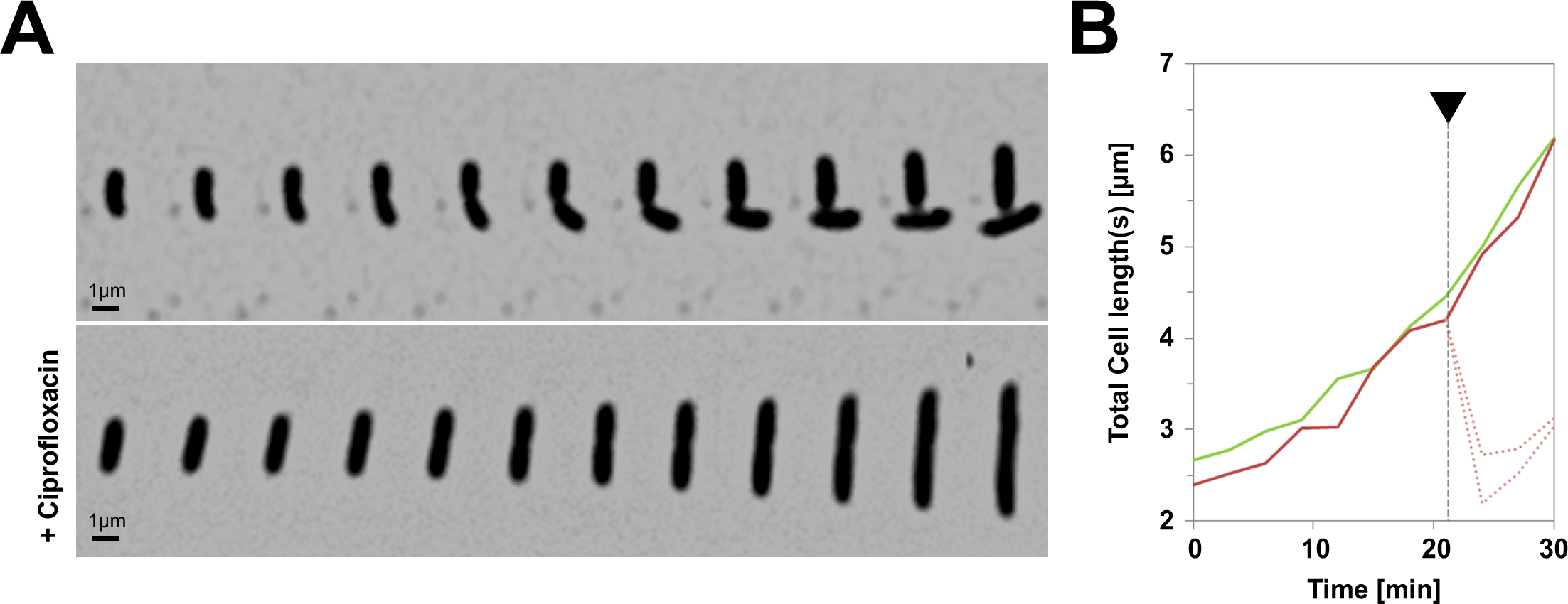

Supplement: Supplementary file 1 [file mbo30002-0123-SD1.tif]

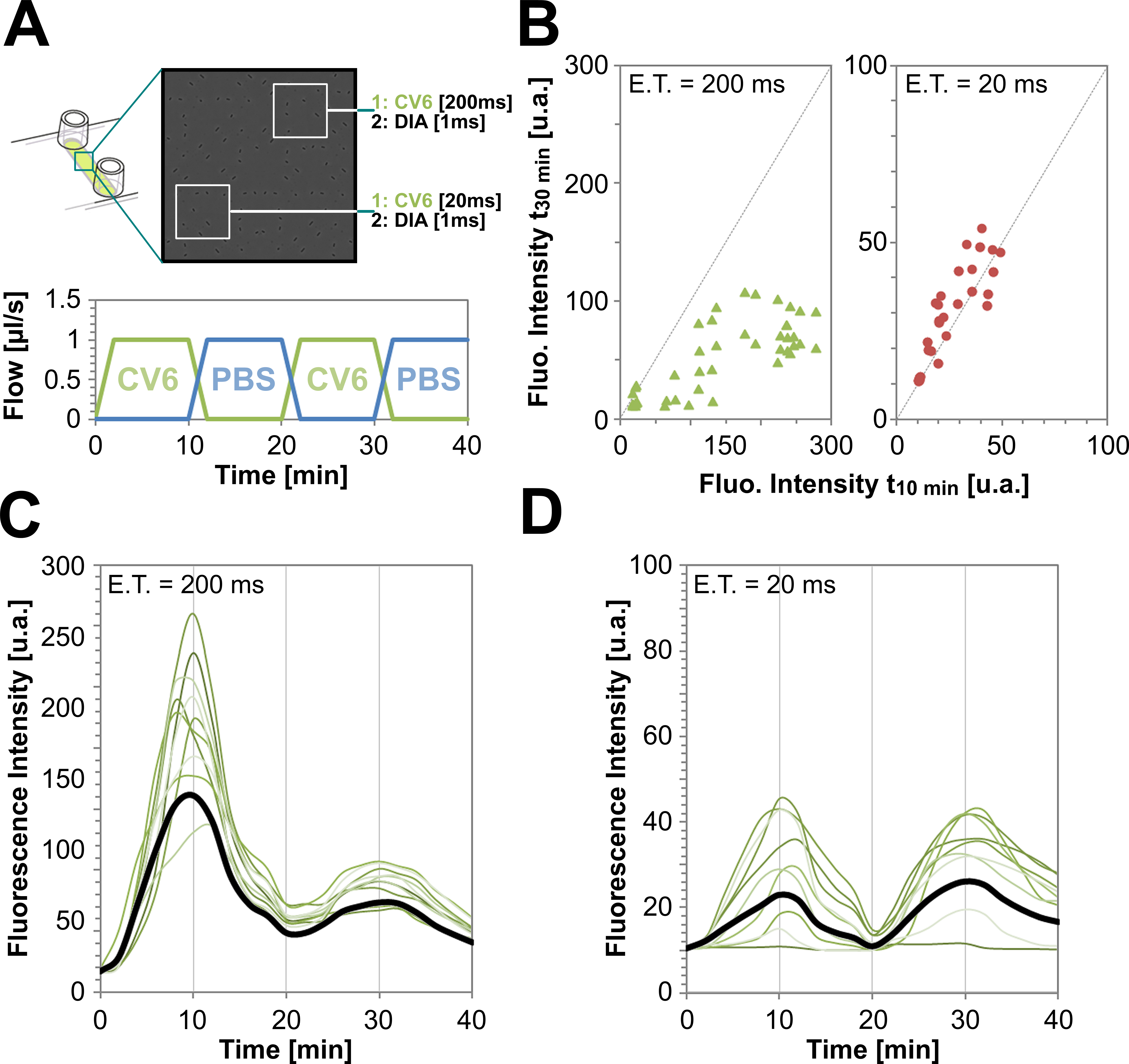

Supplement: Supplementary file 2 [file mbo30002-0123-SD2.tif]

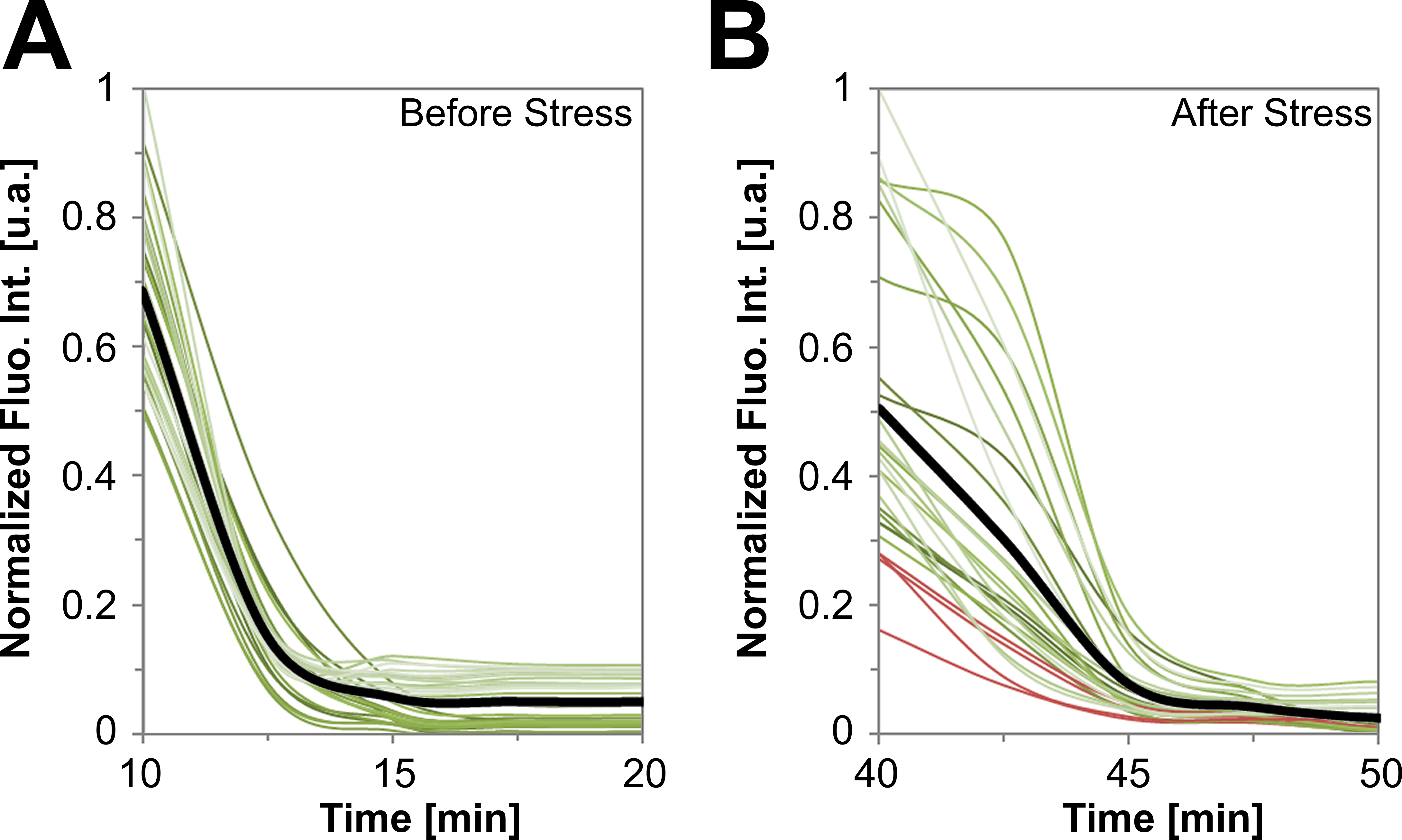

Supplement: Supplementary file 3 [file mbo30002-0123-SD3.tif]
